# Structural Basis of the Function of Yariv Reagent—An Important Tool to Study Arabinogalactan Proteins

**DOI:** 10.3389/fmolb.2021.682858

**Published:** 2021-06-07

**Authors:** Tereza Přerovská, Anna Pavlů, Dzianis Hancharyk, Anna Rodionova, Anna Vavříková, Vojtěch Spiwok

**Affiliations:** ^1^ Department of Biochemistry and Microbiology, University of Chemistry and Technology, Prague, Czechia; ^2^ Department of Informatics and Chemistry, University of Chemistry and Technology, Prague, Czechia

**Keywords:** arabinogalactan proteins (AGPs), Yariv phenylglycoside, molecular dynamics simulation, noncovalent interactions, glycochemistry

## Abstract

Arabinogalactan proteins are very abundant, heavily glycosylated plant cell wall proteins. They are intensively studied because of their crucial role in plant development as well as their function in plant defence. Research of these biomacromolecules is complicated by the lack of tools for their analysis and characterisation due to their extreme heterogeneity. One of the few available tools for detection, isolation, characterisation, and functional studies of arabinogalactan proteins is Yariv reagents. Yariv reagent is a synthetic aromatic glycoconjugate originally prepared as an antigen for immunization. Later, it was found that this compound can precipitate arabinogalactan proteins, namely, their *ß*-D-(1→3)-galactan structures. Even though this compound has been intensively used for decades, the structural basis of arabinogalactan protein precipitation by Yariv is not known. Multiple biophysical studies have been published, but none of them attempted to elucidate the three-dimensional structure of the Yariv-galactan complex. Here we use a series of molecular dynamics simulations of systems containing one or multiple molecules of *ß*-D-galactosyl Yariv reagent with or without oligo *ß*-D-(1→3)-galactan to predict the structure of the complex. According to our model of Yariv-galactan complexes, Yariv reagent forms stacked oligomers stabilized by *π*-*π* and CH/*π* interactions. These oligomers may contain irregularities. Galactan structures crosslink these Yariv oligomers. The results were compared with studies in literature.

## Introduction

Arabinogalactan proteins (AGPs) represent an extremely heterogeneous group of plant cell wall proteoglycans, which together with moderately glycosylated extensins and minimally glycosylated proline-rich proteins belong to the superfamily of hydroxyproline-rich glycoproteins (HRGPs, Showalter et al., 2010). A general feature of all HRGP members is the presence of hydroxylated proline residues, which is a prerequisite for their further glycosylation (Gorres and Raines, 2010; Nguema-Ona et al., 2014). Despite the increasing amount of discovered chimeric or hybrid AGPs, the general characteristics of AGPs were defined over the years. Among them belong the high amounts of Pro, Ala, Ser, and Thr (altogether known as PAST) regularly arranged in Ala-Pro, Ser-Pro and Thr-Pro dipeptide motifs, which governs AGP specific *O*-glycosylation (Tan et al., 2003; Ma et al., 2017). Commonly, the carbohydrate moiety consists of β-D-(1,3)-galactan backbone with β-D-(1,6)-galactan side chains, which are often further substituted by arabinose, rhamnose, fucose, or glucuronic acid (Ellis et al., 2010; Knoch et al., 2014; Ma et al., 2018). Moreover, AGPs contain signal sequences directing them to the extracellular location and often can be found anchored to the plasma membrane by the glycosylphosphatidylinositol (GPI) anchor (Seifert and Roberts, 2007). AGPs are being extensively studied especially in higher plants because they have been shown to play an essential role in plant growth, development, reproduction, signaling, and stress responses (Nguema-Ona et al., 2012, Nguema-Ona et al., 2013; Lamport and Várnai 2013; Olmos et al., 2017; Ma et al., 2018; Su and Higashiyama 2018; Seifert 2020).

There are various tools to study these proteins including monoclonal antibodies, β-Yariv reagents, specific degradation of AGP sugar chains, chemical synthesis, and bioinformatics approach (Su and Higashiyama, 2018). While bioinformatics offers quick and high-throughput analysis, the results reflect only the differences in the protein backbone and no information regarding their glycosylation, the most important part in terms of their function, can be obtained in such a way (Showalter et al., 2010; Johnson et al., 2017; Ma et al., 2017). From the experimental point of view, immunolabeling with antibodies or the use of β-Yariv reagents is the most commonly used (Nguema-Ona et al., 2012). Compared to the monoclonal antibodies, the β-Yariv reagents are able (in addition to the visualization of AGPs) also to perturb their function, which is widely exploited in AGP functional studies (Willats and Knox, 1996; Tang et al., 2006; Nguema-Ona et al., 2007; Yu and Zhao, 2012; Olmos et al., 2017; Su and Higashiyama, 2018; Castilleux et al., 2020).

**FIGURE 1:**
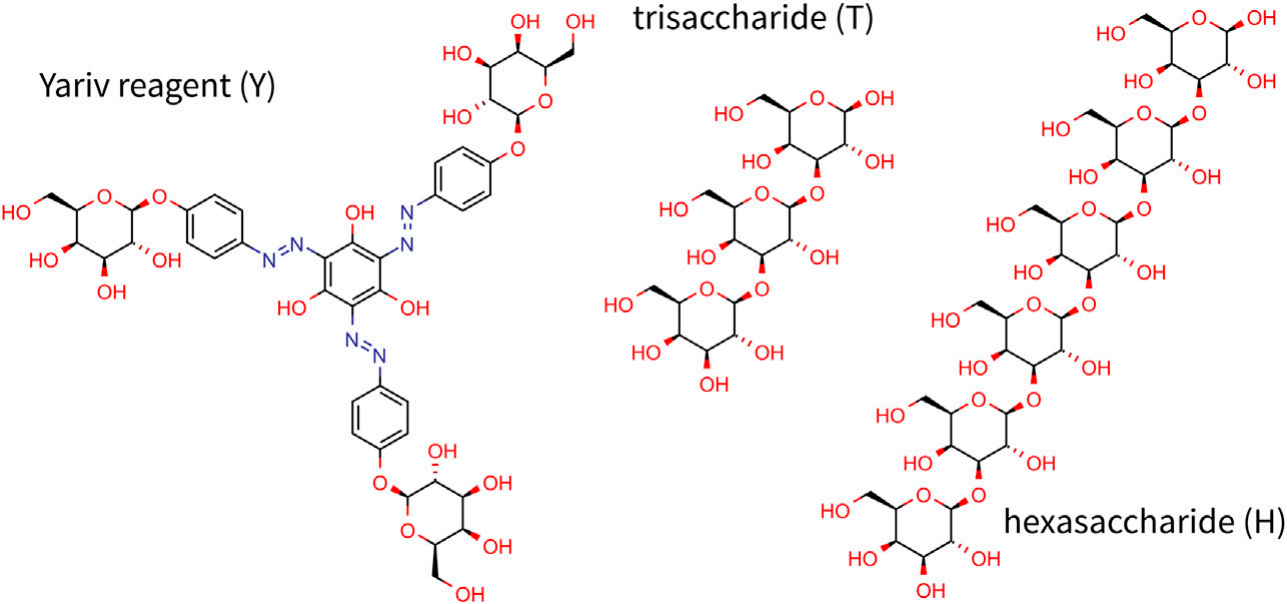
Chemical structure of Yariv reagent and oligosaccharides studied in this study.

Yariv reagents (Figure 1) are synthetic phenylglycosides, which were formerly developed as protein-free precipitatory antigens for determining the content of sugar-binding proteins and their purification (Yariv et al., 1962). Nevertheless, later, certain types of β-Yariv reagents (β-D-glucosyl and β-D-galactosyl) were demonstrated to selectively bind to AGPs (Yariv et al., 1967; Nothnagel 1997). Thus, the ability to bind β-Yariv reagents is also considered a characteristic feature of AGPs. Despite their wide use and attempts to resolve their mode of action, their target structure as well as the mechanism remained elusive for decades (Nothnagel, 1997). Only recently, the target structure has been at least partially clarified. Kitazawa et al. (2013) proved that β-D-galactosyl Yariv reagent interacts with the β-(1,3)-galactan backbone, which has to be longer than five residues for the interaction to occur. Moreover, Sato et al. (2018) showed that the extent of β-(1,6)-galactan substitution affects the Yariv reagent binding ability. Interestingly, the Yariv reagent self-aggregates in the aqueous solution up to approximately 305 units (Nothnagel, 1997; Paulsen et al., 2014). The size of aggregates influences the interaction with AGPs, when the AGP precipitation is known to take place in a solution with ionic strength corresponding to 1% NaCl in which the number of aggregated molecules is approximately 185. On the other hand, 10% NaCl inhibited the precipitation, and the aggregate comprised approximately 125 units (Nothnagel, 1997; Paulsen et al., 2014).

Unfortunately, the mechanism of action still remains largely understudied and more studies are needed to fully understand the nature and functionality of Yariv reagents. To elucidate the structure of Yariv-galactan complexes, we carried out a series of molecular dynamics simulations of systems containing Yariv reagent (namely β-D-galactosyl Yariv, further referred to as Yariv) and/or galactan oligosaccharides in explicitly modeled water. The protein part of AGP was not modeled because it has been shown that Yariv recognizes the carbohydrate part of AGPs (Kitazawa et al., 2013). Galactan trisaccharide was chosen as a minimal model oligosaccharide (Figure 1). This choice was driven by the fact that Yariv is selective for 1→3 linked oligomers, for which a trisaccharide is the smallest representative. Another oligosaccharide studied in this study is hexassacharide (Figure 1) because it was shown that oligosaccharides with more than five units form stable complexes with the Yariv reagent (Kitazawa et al., 2013).

## Materials and Methods

All simulations were done in Gromacs package version 2018 (Abraham et al., 2015). Galactooligosaccharides were modeled using the Glycam 06j-1 force field (Kirschner et al., 2008). Yariv compound was modeled by a manually combined Glycam and General Amber Force Field (Wang et al., 2004). Acpype (Sousa da Silva and Vranken, 2012) was used to convert AMBER files to Gromacs files. Galactose units were modeled by Glycam nonbonded and bonded parameters, except for partial atomic charges. Non-saccharidic part of Yariv compound was modeled by General Amber Force Field in AMBER tools version 16 (Salomon-Ferrer et al., 2013) non-bonded and bonded parameters, except for partial atomic charges. Parameters for the connection between both parts were taken from the analogous parameters of glycoside linkage in Glycam. Partial atomic charges were calculated by an Antechamber routine utilizing the semiempirical AM1-BCC method (Jakalian et al., 2000). Gromacs topology of all studied molecules is available *via* Zenodo (see below).

Systems containing different molecular assemblies were solvated by TIP3P water molecules (Jorgensen et al., 1983). Next, it was minimized by the steepest descent algorithm and equilibrated by 1 ns simulation in a NPT ensemble and 1 ns simulation in a NVT ensemble. This was followed by a 100 ns production simulation. Simulation step was set to 2 fs and all bonds to hydrogens were constrained by the LINCS algorithm (Hess et al., 1997). Electrostatic interactions were modeled by the Particle-Mesh Ewald (PME) (Darden et al., 1993) method with the cutoff set to 1 nm. Temperature and pressure was maintained by Parrinello-Bussi (Bussi et al., 2007) and Parrinello-Raman (Parrinello and Rahman, 1981) algorithm, respectively.

Simulations were analysed using in-house scripts in Python with MDTraj library (McGibbon et al., 2015). Simulation inputs and results (input files for simulations, trajectories without water molecules) are available *via* Zenodo (DOI: 10.5281/zenodo.4767970).

## Results and Discussion

To elucidate the structural organisation of complexes of Yariv reagent with β-D-(1→3)-galactan molecules, we carried out simulations of, in total, 48 systems containing various numbers of Yariv reagent molecules (Y) and carbohydrate molecules. Carbohydrate molecules included trisaccharide (T, β-D-Gal-β-D-(1→3)-Gal-β-D-(1→3)-Gal), or hexasaccharide (H, β-D-Gal-β-D-(1→3)-Gal-β-D-(1→3)-β-D-Gal-β-D-(1→3)-Gal-β-D-(1→3)-β-D-Gal-β-D-(1→3)-Gal). They are further referred to as Y2 for a system with two molecules of Yariv reagent, YT for a system with Yariv reagent and trisaccharide, etc. Initial structures of the systems were assembled manually. In summary, simulated systems included Y2, YT, YH, Y4, Y4T, and Y4H, all in eight replicas.

Our initial simulations (data not shown) showed that noncovalent interactions between a Yariv molecule and a carbohydrate and especially between two Yariv molecules are relatively strong. It would be necessary to run very long simulations to observe relevant structural transitions. Therefore, to map possible modes of interactions between Yariv and carbohydrates we carried out a series of short (100 ns) simulations starting from different initial structures. We believe such simulations are more representative than few long simulations due to long lifetimes of complexes. Initial coordinates were built manually to represent wide diversity in terms of initial distances and orientations of molecules.

First, we were interested in the interactions between two Yariv molecules. The simulated systems of Yariv dimers contained 2073–2083 water molecules. This corresponds to a concentration of Yariv reagent equal to 53–54 mmol/L (approx 28 g/L). This is approximately 30 times higher than concentrations used in precipitation experiments of arabinogalactans, however, in simulations the interactions between Yariv reagent molecules are limited by periodic boundary conditions.

**FIGURE 2:**
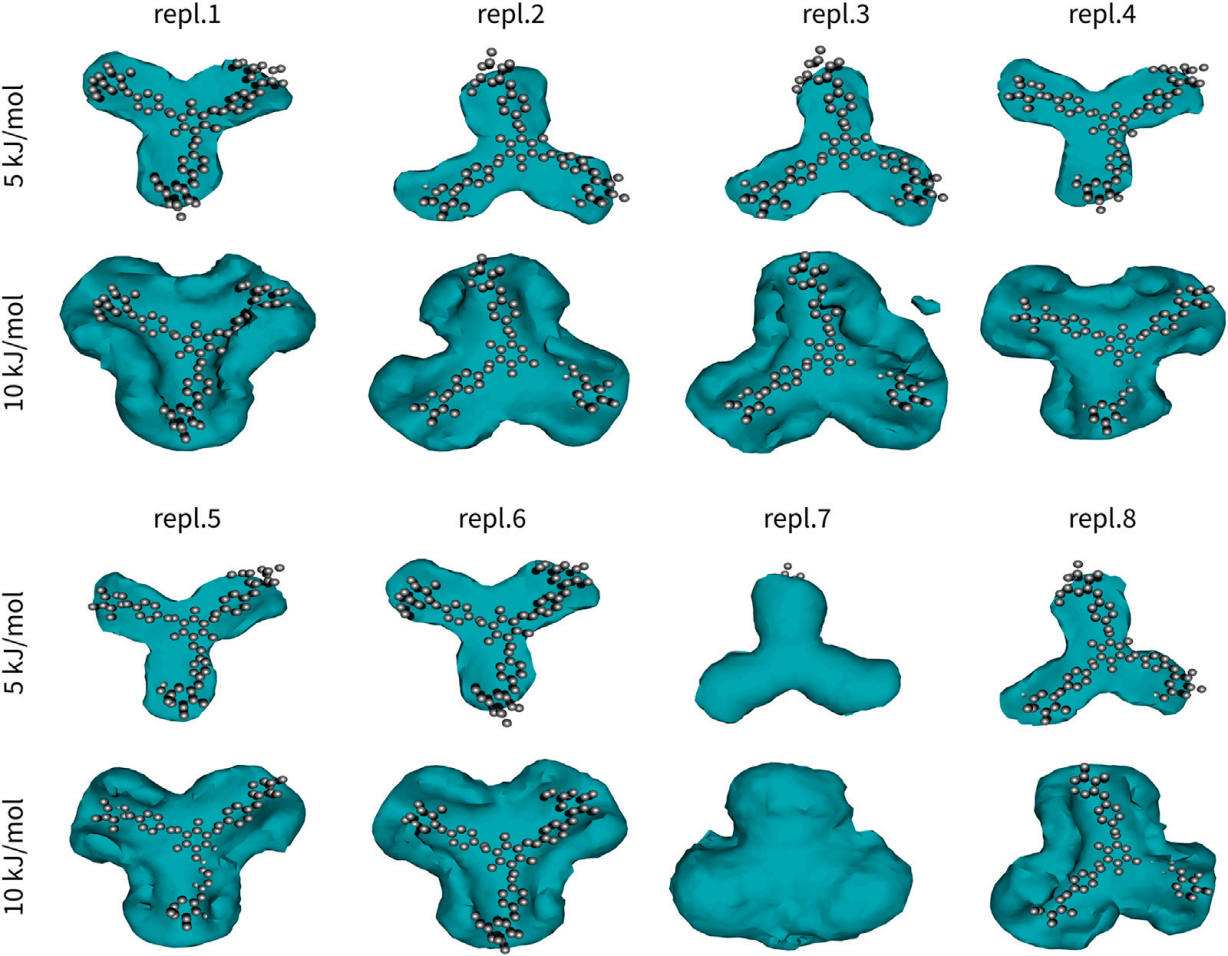
Interactions in Yariv reagent dimer (Y2). One molecule of Yariv was fitted to the initial coordinates. Coordinates of the second molecule were analysed in terms of a free-energy-like function. This function is depicted as isosurfaces at 5 or 10 kJ/mol (relative to the global minimum).

The first Yariv molecule of all snapshots was fitted onto the first snapshot to eliminate its translational and rotational motions. This was possible due to relatively high rigidity of the Yariv molecule (conjugated diazenyl groups). Coordinates of the second molecule were analysed in terms of free-energy-like function. 3D histograms of all carbon atoms of the second Yariv molecule were calculated with 1 Å × 1 Å × 1 Å bins. Next, these values were converted to free-energy-like functions as:

$$A_{i}=-kT\text{log}P_{i}$$

where *P*_*i*_ is the histogram count, *k* is Boltzmann constant, and *T* is the temperature in Kelvin. Finally, the value of the global minimum was subtracted. The difference between a free energy and the free-energy-like function used in this study is in the fact that the free energy depicts probability of finding a molecule at a certain point, whereas the free-energy-like function depicts the probability of finding any carbon atom at a certain point. The advantage of the free-energy-like function is in its higher resolution. The resulting free-energy-like functions are depicted in Figure 2.

Yariv reagent formed stable and almost perfectly parallel dimers (Figure 2). Two Yariv reagent molecules interact by *π*-*π* stacking *via* all four aromatic moieties, despite different initial monomer orientations. These interactions are relatively strong, but still reversible as indicated by replica seven. In this simulation replica, we observed that the second Yariv molecule (initially at the bottom, depicted as a free-energy-like function in Figure 2) migrated to the top face of the first Yariv molecule (depicted as atoms in Figure 2). Interestingly, assemblies stabilized by CH/*π* interactions between the carbohydrate part of one Yariv molecule and the aromatic ring in the second molecule were rare. This can be explained by the fact that galactose forms aromatic CH/*π* interactions in carbohydrate-protein complexes *via* its C-H bonds on carbons C3, C4, C5, and C6. Such complexes are not parallel. In contrast, glucose forms parallel aromatic CH/*π* complexes in carbohydrate-protein complexes *via* C-H bonds on carbon atoms C1, C3, and C5 or C2, C4, and C6. In conclusion, Yariv forms dimers that are parallel and stabilized predominantly by *π*-π stacking between its aromatic moieties. This assembly is relatively stable, nevertheless rearrangements of the assembly are possible in sub-microsecond time scales.

**FIGURE 3:**
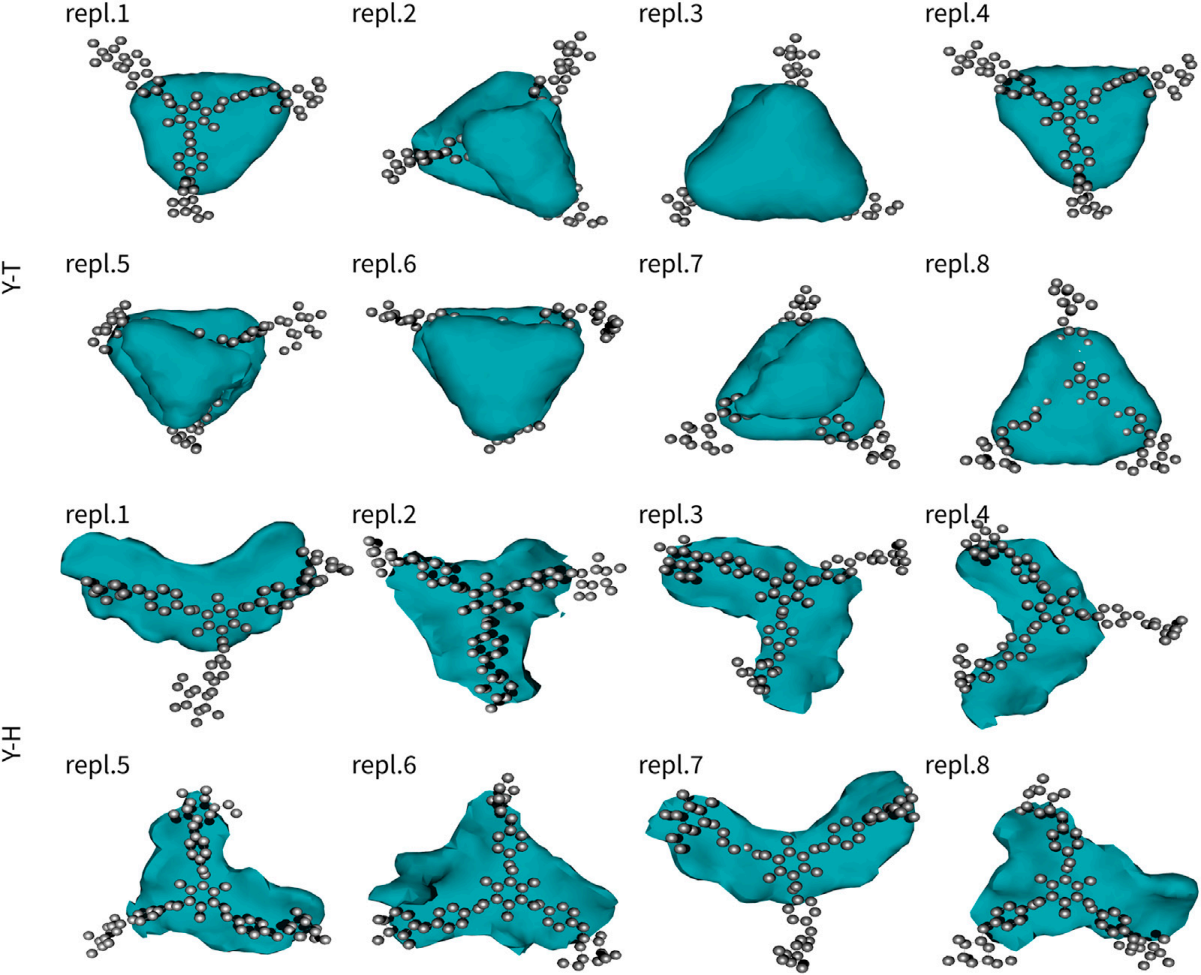
Interactions of Yariv reagent with trisaccharide (YT) and hexasaccharide (YH). The molecule of Yariv was fitted to the initial coordinates. Coordinates of the saccharide molecule were analysed in terms of a free-energy-like function. This function is depicted as isosurfaces at 5 kJ/mol (relative to the global minimum).

The second series of simulations studied assemblies of a single Yariv reagent with a single trisaccharide or hexasaccharide. Similarly to Y2, also these results were analysed in terms of free-energy-like functions (Figure 3). Similarly to Yariv dimers, the complexes of Yariv reagent with oligosaccharides were stable. We observed the migration of trisaccharide from the bottom to the top face of Yariv in five of eight 100-ns-long simulations. Galactooligosaccharides with β-(1→3) linkage are characterized by approximately 120° angle between three adjacent monosaccharide units. They are perfectly aligned with the orientation of aromatic rings (*peripheral*-*central*-*peripheral*) in a Yariv reagent molecule. Yariv-galactooligosaccharide complexes were stabilized by carbohydrate-aromatic CH/*π* interactions (Spiwok, 2017). Free-energy-like functions of YT complexes were triangular. This can be explained by the formation of three possible complexes in which the three adjacent monosaccharide units interact either with *peripheral1*-*central*-*peripheral2*, *peripheral2*-*central*-*peripheral3*, or *peripheral3*-*central*-*peripheral1* aromatic rings. Fast interconversion between these complexes determines the triangular free-energy-like functions of YT complexes.

Complexes of Yariv reagent with hexasaccharide (YH) were comparably strong as YT. Free-energy-like functions were mostly boomerang-shaped (five of eight simulations). This can be explained by the fact that the interconversion between complexes was much slower compared to YT and one assembly was predominant.

**FIGURE 4:**
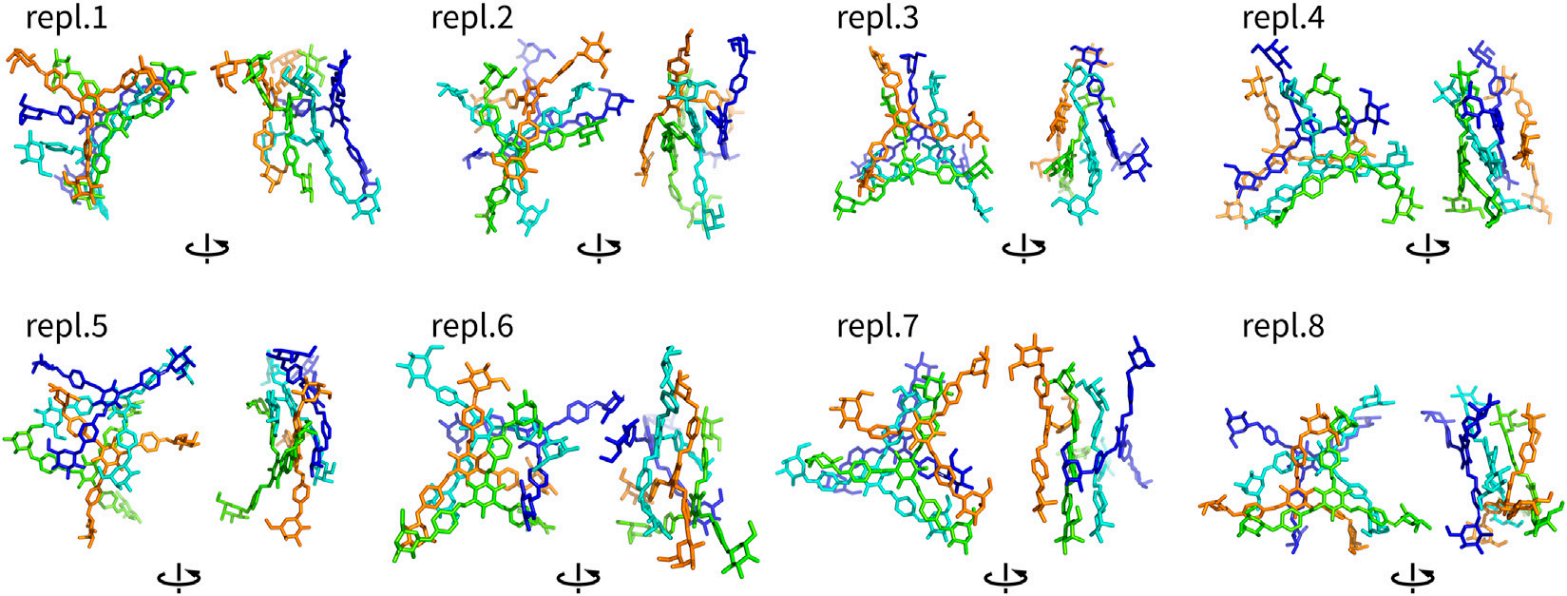
Assemblies Y4 (non-hydrogen atoms) after 100 ns shown in two different orientations rotated by 90°. They are colored by atom ID in the system.

**FIGURE 5:**
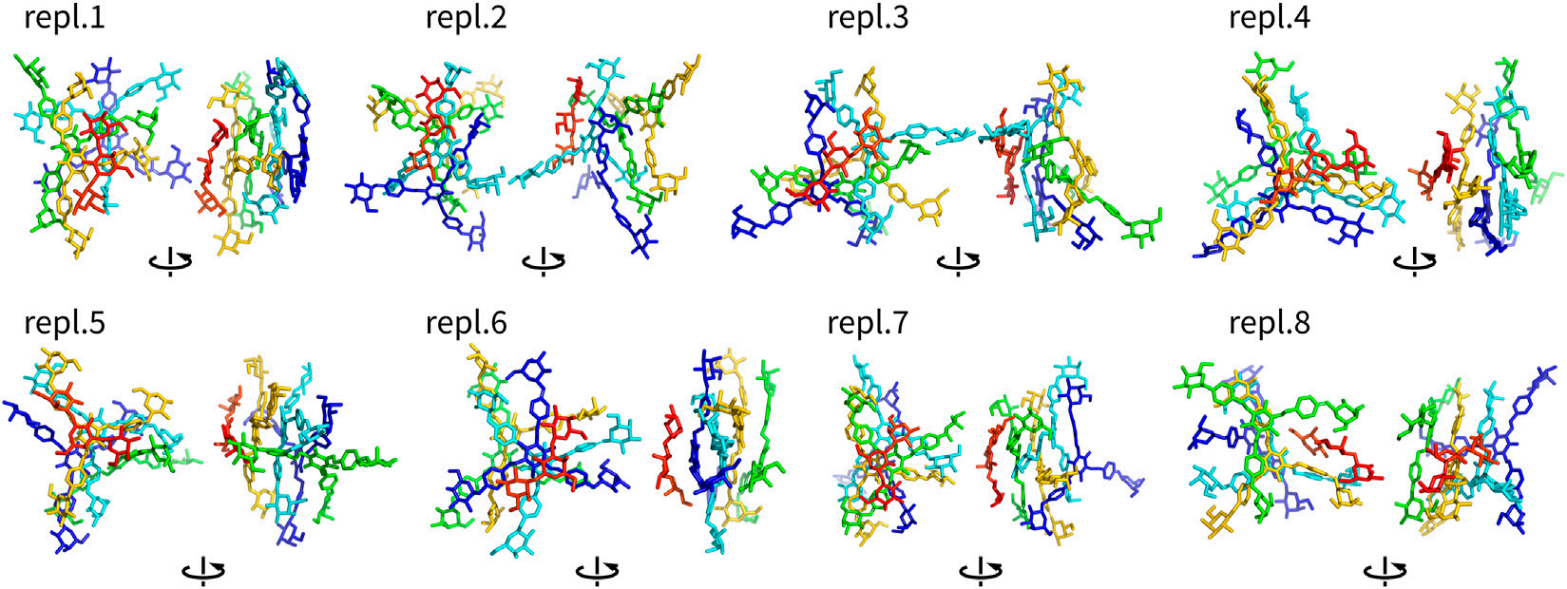
Assemblies Y4T (non-hydrogen atoms) after 100 ns shown in two different orientations rotated by 90°. They are colored by atom ID in the system (Yariv molecules are blue to orange, saccharides are in red).

**FIGURE 6:**
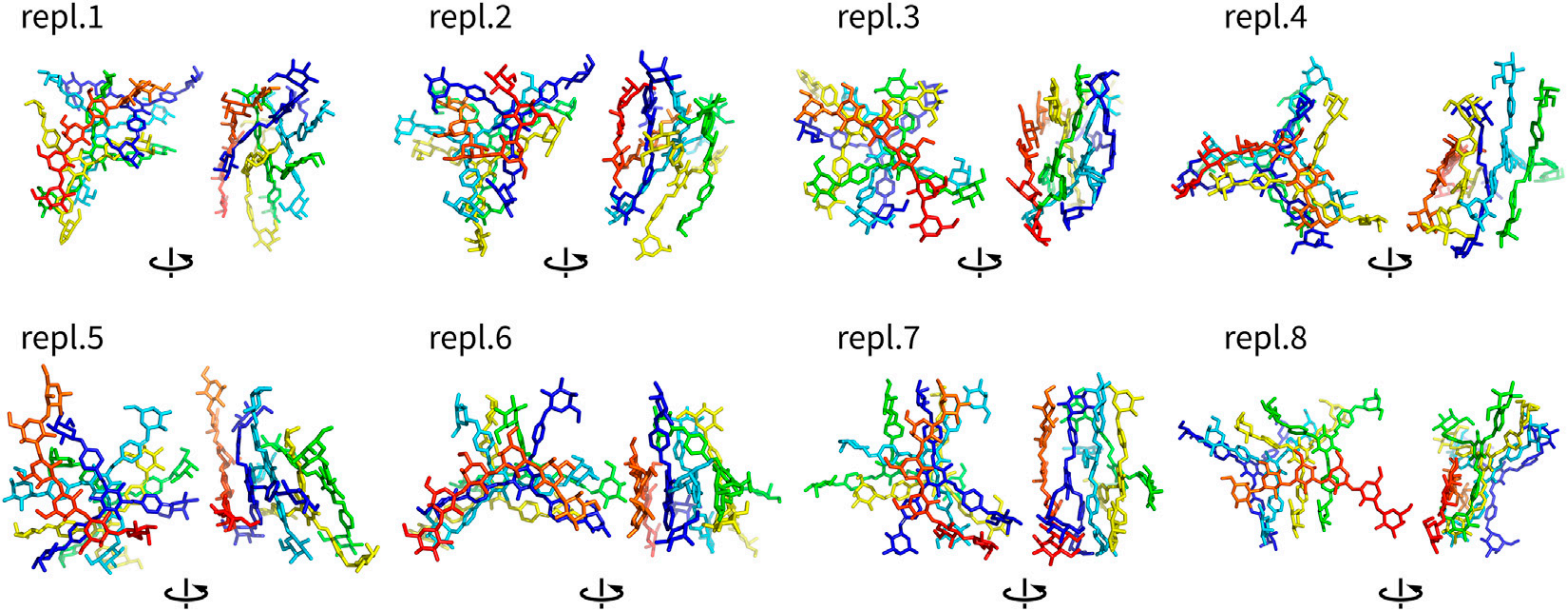
Assemblies Y4H (non-hydrogen atoms) after 100 ns shown in two different orientations rotated by 90°. They are colored by atom ID in the system (Yariv molecules are blue to orange, saccharides are in red).

Assemblies with more than two molecules included Y4, Y4T, and Y4H. The complexes formed in these systems were visualized as structures after 100 ns (Figures 4–6). These complexes were formed very quickly (<20 ns) and they were stable in terms of topology along 100 ns simulations. These figures show that Yariv reagent molecules were arranged in parallel, however, these assemblies were not perfectly parallel and contained numerous irregularities. Yariv molecules interacted predominantly *via π*-*π* stacking of their aromatic molecules. There were also CH/*π* interactions between the carbohydrate part of one Yariv molecule and an aromatic ring of another molecule. Furthermore, there were CH/*π* interactions in which aromatic rings were playing both roles—donors and acceptors.

Complexes of multiple Yariv molecules with a saccharide (Figures 5, 6) combined the properties of Yariv tetramers and binary complexes described above. The complexes were formed by a parallel assembly of Yariv molecules with irregularities from a perfectly parallel shape. A molecule of tri- or hexasaccharide was sitting on top of the Yariv molecule which was most exposed to the solvent. Binding of saccharides onto Yariv was slightly different from that in binary complexes. This can be explained by the fact that there are irregularities in the parallel shape of Yariv tetramers. These irregularities expose more aromatic groups for interaction with a saccharide and give saccharides more freedom.

One exception was the Y4H complex formed in the replica eight. In this case the oligosaccharide molecule docked into the groove formed by two peripheral and one central aromatic moieties of parallely stacked Yariv tetramer. This complex is stabilized by numerous hydrogen bonds. This assembly can be seen as an alternative model of Yariv-oligosaccharide interactions.

It is important to assess the accuracy of the simulations in this study. This accuracy is determined by the accuracy of molecular mechanics potentials (force fields) and by the completeness of sampling. The studied complexes were dominated by *π*-*π* and CH/*π* interactions. Comparison of quantum chemical and molecular mechanical energies of sample *π*-π (Sponer et al., 2006) or CH/*π* (Spiwok et al., 2005) complexes has shown that these interactions are relatively accurately modeled by the available molecular mechanics force fields. Another issue in carbohydrate modeling is ring puckering. Hexopyranoses may exist in the chair as well as in the boat or skew-boat conformers. The chair structure is predominant for β-D-galactose units. Visual inspection of trajectories revealed that carbohydrates stayed in the chair conformation as expected.

Finally, molecular dynamics simulation suffers limited time scales due to its computational complexity. Here we used multiple replicas of simulated systems differing in the initial structure of the systems rather than running a few long simulations. This was motivated by the necessity to map possible interaction patterns.

**FIGURE 7:**
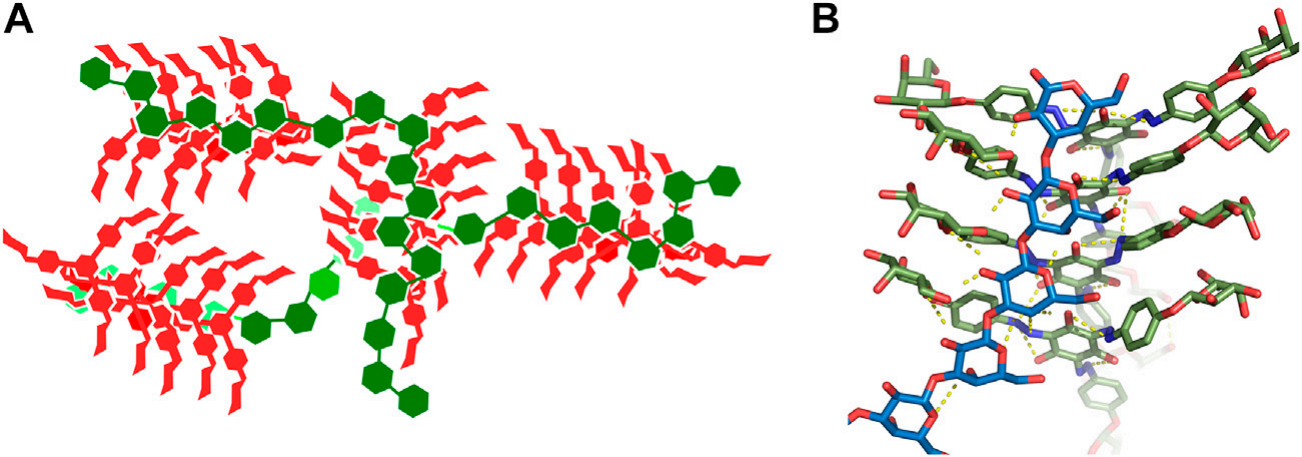
**(A)**-Schematic view of a model of a complex of Yariv reagent with *ß*-(1→3)-galactans. Yariv is in red, saccharides are in green. **(B)**-Detailed view of the Y4H complex in replica eight.

Our model of complexes of Yariv reagent with β-(1→3)-galactans is depicted in Figure 7A as a schematic view. It is our speculation of the structure of large Yarive-polysaccharide complexes based on the results of our simulations. Yariv forms parallel stacked oligomers. The sizes of these oligomers may vary, but we expect their size in tens or hundreds of units. In simulations of Yariv dimers, we observed a trend of rotation of its units, i.e., one unit is rotated by a few degrees. This rotation seems to be asymmetric (right handed). We speculate that this may explain the helical chirality of Yariv aggregates that has been observed by circular dichroism (Hoshing et al., 2020).

These stacked oligomers are not perfect and contain irregularities. This is probably the reason Yariv reagent and its complexes resisted the application of conventional experimental methods. Some experimental methods of structure elucidation, such as crystallography, require strong periodicity of the studied system. Irregularities in oligomeric structures provide more accessible aromatic rings as platforms for interaction with carbohydrates.

An assembly that cannot be ruled out as a model of Yariv-oligosaccharide interaction is the one formed in the eighth replica of Y4H simulation (Figure 6 and Figure 7B in detail). We plan to study this assembly in future.

The selectivity of Yariv reagent towards 1→3-linked oligo- and polysaccharides may be explained by the fact that the three adjacent aromatic rings in Yariv and three adjacent monosaccharide units in 1→3-linked oligosaccharide are bent by 120°. For example, in 1→4-linked glycans, the orientation is linear (not bent) and such oligo- or polysaccharide would bind to the Yariv reagent very weakly.

The fact that Yariv complexes are stabilized mostly by *π*-*π* and CH/*π* interactions is in good agreement with the fact that Yariv assemblies are resistant to high ionic strength (Nothnagel, 1997; Paulsen et al., 2014). These interactions are mediated by the hydrophobic parts of both molecules, namely, by aromatic rings and C-H-rich patches of carbohydrates. Due to this, many researchers present these interactions as hydrophobic. It was not a subject of this work to determine whether these interactions are physical attractive interactions or a result of solvation and desolvation. The nature of, for example, CH/*π* interactions remains a question of debate (physical van der Waals vs. hydrophobic) (Spiwok, 2017).

In conclusion, simulations of systems containing the Yariv reagent with model oligosaccharides provide predictions of main interaction types and structural arrangements in these complexes. We understand that our models are based on simplified systems and short time scales, nevertheless, we believe they can inspire other researchers studying the Yariv reagent to design new biophysical experiments or Yariv derivatives to complete our picture of the function of this useful reagent.

## Data Availability

The datasets presented in this study can be found in online repositories. The names of the repository/repositories and accession number(s) can be found below: https://zenodo.org, DOI: 10.5281/zenodo.4767970.
